# A family of functional dissimilarity measures for presence and absence data

**DOI:** 10.1002/ece3.2214

**Published:** 2016-07-05

**Authors:** Carlo Ricotta, János Podani, Sandrine Pavoine

**Affiliations:** ^1^ Department of Environmental BiologyUniversity of Rome “La Sapienza”Piazzale Aldo Moro 500185RomeItaly; ^2^ Department of Plant Systematics, Ecology and Theoretical BiologyMTA‐ELTE‐MTM Ecology Research GroupInstitute of BiologyL. Eötvös UniversityPázmány P. s. 1/CH‐1117BudapestHungary; ^3^ Centre d'Ecologie et des Sciences de la Conservation (CESCO UMR7204)Sorbonne Universités, MNHN, CNRS, UPMCCP51, 55‐61 rue BuffonParisFrance

**Keywords:** Between‐species dissimilarities, contingency table, functional excess, functional turnover, species richness

## Abstract

Plot‐to‐plot dissimilarity measures are considered a valuable tool for understanding the complex ecological mechanisms that drive community composition. Traditional presence/absence coefficients are usually based on different combinations of the matching/mismatching components of the 2 × 2 contingency table. However, more recently, dissimilarity measures that incorporate information about the degree of functional differences between the species in both plots have received increasing attention. This is because such “functional dissimilarity measures” capture information on the species' functional traits, which is ignored by traditional coefficients. Therefore, functional dissimilarity measures tend to correlate more strongly with ecosystem‐level processes, as species influence these processes via their traits. In this study, we introduce a new family of dissimilarity measures for presence and absence data, which consider functional dissimilarities among species in the calculation of the matching/mismatching components of the 2 × 2 contingency table. Within this family, the behavior of the Jaccard coefficient, together with its additive components, species replacement, and richness difference, is examined by graphical comparisons and ordinations based on simulated data.

## Introduction

Dissimilarity coefficients between pairs of species assemblages (communities, plots, relevés, sites, quadrats, etc.) provide a helpful tool for exploring the complex ecological mechanisms that drive community assembly. Most of these measures summarize different facets of plot‐to‐plot dissimilarity based either on species presence/absence scores or on species abundances, thus implicitly assuming that all species are equally and maximally distinct from one another (Ricotta et al. 2015).

In particular, given two plots *X* and *Y*, dissimilarity coefficients for presence and absence data are usually formulated using the matching/mismatching components *a*,* b*, and *c* of a 2 × 2 contingency table: *a* is the number of species present in both plots, *b* is the number of species present only in plot *X*, and *c* is the number of species present only in plot *Y*, such that *a* + *b* + *c* is the total number of species in the two plots. Using the values of *a*,* b*, and *c*, a large number of coefficients can be calculated, such as the Jaccard (1900) index of dissimilarity *J* = (*b* + *c*)/(*a* + *b* + *c*), or the Sørensen (1948) index *S* = (*b* + *c*)/(2*a* + *b* + *c*). For a comprehensive inventory of presence/absence dissimilarity coefficients, see Podani (2000) and Legendre and Legendre (2012). A common feature of most presence/absence indices is that they are usually expressed in terms of a ratio where the numerator, that is the “operational part” of the index *sensu* Legendre (2014), estimates the amount of plot‐to‐plot dissimilarity depending on the purpose of the study, while the denominator scales the index to values between 0 and 1. Note that the fourth component of the contingency table *d*, which represents joint absences (i.e., the species absent from both plots being compared but found in other plots), is only rarely used in community ecology for the calculation of dissimilarity measures. Therefore, in this study we will not discuss dissimilarity coefficients, which include mutual absences in their formulation.

More recently, dissimilarity measures that incorporate information about the degree of functional differences between the species in both assemblages have received considerable attention (Izsák and Price 2001; Champely and Chessel 2002; Pavoine et al. 2004; Ricotta and Bacaro 2010; Chiu and Chao 2014; Chiu et al. 2014; Pavoine and Ricotta 2014; Ricotta and Pavoine 2015). This is because such “functional dissimilarity measures” capture information on the species' functional traits, which is ignored by traditional dissimilarity measures. Functional traits are morphological, physiological, and phenological attributes, which impact individual fitness via their effects on growth, reproduction, and survival (Violle et al. 2007). Therefore, measures of functional dissimilarity tend to correlate more strongly with ecosystem‐level processes, such as productivity, regulation of biogeochemical fluxes, or resilience to perturbations, as species influence these processes via their traits (Mason and de Bello 2013).

The observed relationship between functional dissimilarity and ecosystem functioning raises the question of how to measure functional dissimilarity in meaningful ways. As ecological data are often multivariate with high dimensionality, no single measure summarizes adequately all aspects of functional dissimilarity. Thus, in order to assess relevant differences in the functional organization of species assemblages, a multifaceted approach is needed. While the notion of functional dissimilarity is independent of any particular method of measurement, a number of basic criteria have been proposed that functional dissimilarity measures should meet to behave reasonably in ecological research (e.g., Pavoine and Ricotta 2014). In this study, we first briefly summarize such basic requirements. Then, we introduce a new family of dissimilarity measures for presence and absence data, which consider functional dissimilarities among species in the calculation of the matching/mismatching components of the 2 × 2 contingency table. These presence/absence measures may be useful whenever data on species abundances are either unknown or irrelevant, for example, for large‐scale environmental protection purposes. In these circumstances, presence/absence measures represent the adequate choice for quantifying the functional dissimilarity among plots.

## Methods

Regardless of any specific measure of dissimilarity, in most cases the information available for summarizing the functional organization of a given set of plots is an *N* × *K* matrix of species presences and absences (i.e., usually 0/1 scores) for *N* species and *K* plots, together with an *N* × *τ* matrix with values for *τ* selected functional traits for each species. As most plot‐to‐plot functional dissimilarity indices are built on pairwise functional dissimilarities between species, this latter matrix is first transformed to an *N* × *N* matrix of pairwise functional dissimilarities *d*_*ij*_ between species *i* and *j* in the interval 0–1 with *d*_*ii*_ = 0 and *d*_*ij*_ = *d*_*ji*_. Note that for any dissimilarity index *d*_*ij*_ falling into the unit range, its similarity counterpart *s*_*ij*_ can be simply calculated as *s*_*ij*_ = 1 − *d*_*ij*_, so that *s*_*ij*_ + *d*_*ij*_ = 1.

In this context, to coherently frame the notion of plot‐to‐plot dissimilarity, many authors have proposed a number of basic requirements that a good index should meet to reasonably behave in ecological research (see Anderson et al. 2006; Clarke et al. 2006; Legendre and De Cáceres 2013; Pavoine and Ricotta 2014). Among them, the primary requirements that are generally accepted as necessary for a meaningful (functional) plot‐to‐plot dissimilarity index *D* in the range 0–1 are related to its extreme values: (1) For two identical assemblages, *D* takes the value zero, denoting maximum similarity, and (2) *D* takes the value one, denoting maximum dissimilarity, only for two completely distinct assemblages. This latter criterion is satisfied for assemblages with no species in common and with zero functional similarities between the species in the first assemblage and those in the second assemblage (see, e.g., Pavoine and Ricotta 2014).

While both requirements are straightforward, they have a rich corollary of implications that may help in generalizing traditional presence/absence dissimilarity measures among plots to include functional dissimilarities among species. For instance, requirement (2) implies that the dissimilarity component of the generalized index depends on how the dissimilarities between the species in the first assemblage and those in the second assemblage are distributed. Accordingly, in the calculation of a generalized index *D* of functional dissimilarity between two plots *X* and *Y*, the traditional mismatching components *b* and *c* of the 2 × 2 contingency table can be expressed as follows to include functional differences among species:

(1) $$B=\sum_{i\in X,i\notin Y}\min_{j\in Y}\left\{d_{ij}\right\}$$

and

(2) $$C=\sum_{j\in Y,j\notin X}\min_{i\in X}\left\{d_{ij}\right\}$$

where *B* and *C* are the generalized counterparts of the traditional mismatching components *b* and *c* and the summations in equations (1) and (2) are taken over all species that are present only in plot *X* or *Y*, respectively (i.e., the species contributing to *b* and *c* of the contingency table). The components *B* and *C* thus represent the functional uniqueness of plot *X* compared with plot *Y* and vice versa.

Among the possible options for calculating how different a species of one assemblage is from the species of the other assemblage, the minimum functional dissimilarity (see, e.g., Izsák and Price 2001) has been chosen. Accordingly, for the calculation of the component *B* we used the minimum functional dissimilarity $\min_{j\in Y}\left\{d_{ij}\right\}$ between species *i* found only in plot *X* and all species in plot *Y*. Likewise, for the calculation of the component *C* we used the minimum functional dissimilarity $\min_{i\in X}\left\{d_{ij}\right\}$ between species *j* found only in plot *Y* and all species in plot *X*. This choice is in agreement with the core of requirement (1) that a species in plot *X* should be compared to its functional nearest neighbor in plot *Y* such that for two identical assemblages the index takes the value zero. Taking the minimum functional dissimilarity also ensures that, given two plots with no species in common but for which each species in plot *X* has a functionally similar species in plot *Y*, the dissimilarity between *X* and *Y* is close to zero. Also, if a species that appears only in plot *X* is functionally identical to a species shared by *X* and *Y*, then its contribution to *B* is zero.

The quantity $\min_{j\in Y}\left\{d_{ij}\right\}$ can be interpreted as the (functional) fraction of species *i* that is not shared by the species in plot *Y*, such that 0 ≤ *B* ≤ *b* and 0 ≤ *C* ≤ *c*. According to equation (1), *B* = 0 if all species that are present only in plot *X* are functionally identical to at least one species in *Y*. At the other extreme, *B* = *b* if all species that are present only in plot *X* are maximally dissimilar from every species in *Y*.

Note that, due to the relationship *s*_*ij*_ = 1 − *d*_*ij*_, the generalized components *B* and *C* can also be expressed as: $B=\sum_{i\in X,i\notin Y}\min_{j\in Y}\left\{1-s_{ij}\right\}=b-\sum_{i\in X,i\notin Y}\max_{j\in Y}\left\{s_{ij}\right\}$ and $C=c-\sum_{j\in Y,j\notin X}\max_{i\in X}\left\{s_{ij}\right\}$, where $\max_{j\in Y}\left\{s_{ij}\right\}$ is the maximum functional similarity between species *i* found only in plot *X* and all species in plot *Y*.

Based on the above definitions, virtually all presence/absence dissimilarity coefficients expressed as a normalized ratio D=Operational Part/Scaling Factor can be generalized to include functional differences among species by substituting the traditional mismatching components *b* and *c* with their generalized counterparts *B* and *C* in the operational part of the index; for example, the generalized expression of the Jaccard dissimilarity takes the following form:

(3) $$J=\frac{B+C}{a+b+c}$$

while the generalized expression of the Sørensen index becomes:

(4) $$S=\frac{B+C}{2a+b+c}$$

The generalized Jaccard dissimilarity can be interpreted as the functional fraction of species in *X* and *Y* that is not shared by the two plots being compared. Note that in equations (3) and (4) the scaling factors (denominators) are the same as in the traditional presence/absence measures. In this way, the generalized dissimilarities conform to requirement (2) that the indices take their maximum value only for two completely distinct assemblages for which *B* = *b* and *C* = *c*. In this case, equations (3) and (4) recover the traditional expressions of the Jaccard and the Sørensen dissimilarity, respectively.

The differences $b-B=\sum_{i\in X,i\notin Y}\max_{j\in Y}\left\{s_{ij}\right\}$ and $c-C=\sum_{j\in Y,j\notin X}\max_{i\in X}\left\{s_{ij}\right\}$ can be interpreted as the functional fraction of species present only in plot *X* that is shared by the species in plot *Y*, and vice versa. Therefore, both terms increase the similarity between *X* and *Y*, such that the generalized counterpart of the traditional matching component *a* of the 2 × 2 contingency becomes

(5) $$A=a+\left(b-B\right)+\left(c-C\right)$$

which ensures that *A* ≥ *a* and *A* + *B* + *C* = *a* + *b* + *c*, meaning that the generalized matching/mismatching components *A*,* B*, and *C* can be expressed in terms of functional species, or functional richness sensu Villéger et al. (2008). According to equation (5), the similarity complement of the Jaccard index can be thus expressed as 1 − *J *= *A*/(*a* + *b* + *c*).

## Worked Example

The potential of the proposed approach for highlighting the relationships between community composition and ecosystem functioning was examined by comparing the generalized Jaccard dissimilarity coefficient (Eq. (3)) with its traditional presence/absence counterpart in virtual plant communities along a simulated ecological gradient. The artificial data of Ricotta and Pavoine (2015) for 15 species (*S1–S15*) and 9 plots (*P1–P9*) were converted to presence/absence scores (Table 1; see also Appendix S1). The corresponding matrix of pairwise functional dissimilarities *d*_*ij*_ between all species pairs is given in Appendix S2. The original species × plots matrix was generated with unimodal response of all species to a one‐dimensional gradient with varying amplitude (length) and intensity (abundance), while the dissimilarity matrix was constructed such that the interspecies dissimilarities reflect the species ecological differences along the simulated gradient in Table 1. For details, see Ricotta and Pavoine (2015).

**Table 1:** Artificial data matrix composed of 15 species (*S1–S15*) and 9 plots (*P1–P9*) for the graphical comparison of the indices used in the worked example. The data are the same as in Ricotta and Pavoine (2015) converted to presence/absence scores. Species presences are highlighted in gray

Species	Plots								
	P1	P2	P3	P4	P5	P6	P7	P8	P9
**S1**	**1**	**1**	0	0	0	0	0	0	0
**S2**	**1**	**1**	0	0	0	0	0	0	0
**S3**	**1**	**1**	**1**	0	0	0	0	0	0
**S4**	**1**	**1**	**1**	**1**	0	0	0	0	0
**S5**	0	**1**	0	0	0	0	0	0	0
**S6**	0	0	**1**	0	0	0	0	0	0
**S7**	**1**	**1**	**1**	**1**	**1**	**1**	0	0	0
**S8**	0	**1**	**1**	**1**	**1**	**1**	0	0	0
**S9**	**1**	**1**	**1**	**1**	**1**	**1**	**1**	0	0
**S10**	0	0	0	**1**	**1**	**1**	0	0	0
**S11**	0	0	0	0	0	**1**	0	0	0
**S12**	0	0	0	0	0	0	**1**	**1**	**1**
**S13**	0	0	0	0	**1**	**1**	**1**	**1**	**1**
**S14**	0	0	0	0	0	0	**1**	**1**	**1**
**S15**	0	0	0	0	0	0	0	**1**	**1**

To highlight the behavior of both indices (traditional vs. generalized Jaccard index), we used profile diagrams: For each index, we calculated the dissimilarity of plot *P1* with itself and with the remaining plots. This operation provides nine dissimilarity values whose graphical illustration shows the effect of changes in community composition along the simulated gradient.

In addition, it has been recently argued that the overall dissimilarity between two species assemblages is actually driven by two different processes, as species assemblages can differ in richness (i.e., one assemblage has more species than the other) and composition (i.e., some species are replaced by others; Baselga 2010). Therefore, we used the additive decomposition proposed by Podani and Schmera (2011) for partitioning the Jaccard dissimilarity coefficient into species replacement (or turnover *J*_Repl_) and richness difference (*J*_Rich_) such that *J* = *J*_Repl_ + *J*_Rich_. The turnover component *J*_Repl_ summarizes how many species in one plot are replaced by a different species in the other plot, normalized by the total species richness of both plots (a+b+c). As one replacement always involves two species (Carvalho et al. 2013), the total number of replaced species is $2\min\left\{b,c\right\}$. Hence, $J_{\mathrm{Repl}}=2\min\left\{b,c\right\}/\left(a+b+c\right)$. By contrast, *J*_Rich_ summarizes the difference in species richness between both plots, normalized in the same way, such that $J_{\mathrm{Rich}}=\left|b-c\right|/\left(a+b+c\right)$. Therefore, in this study we also compared the additive components of the classical Jaccard dissimilarity for presence and absence data with their functional analogues $J_{\mathrm{Repl}}=2\min\left\{B,C\right\}/\left(a+b+c\right)$ and $J_{\mathrm{Rich}}=\left|B-C\right|/\left(a+b+c\right)$. In this case, *J*_Repl_ corresponds to the amount of functional richness unique to site *X* that is replaced by the functional richness at site *Y*, or functional turnover. Likewise, the generalized version of *J*_Rich_ represents the difference in functional richness between plots *X* and *Y*, or, in other words, functional excess.

All calculations were performed with the new R scripts available in Appendix S3 (see Appendix S4 for a guide through the R scripts).

The entire dissimilarity matrices among plots were also subjected to principal coordinates analysis (PCoA) to obtain a compositional ordination with the traditional Jaccard index and a functional ordination with the generalized Jaccard index. Comparison of the two results may give insight into the mechanisms by which a traditional ordination based on compositional dissimilarities only is changed if mismatches are represented by functional dissimilarities between species. The ordinations were calculated by the SYN‐TAX 2000 package (Podani 2001).

## Results

**Figure 1:**
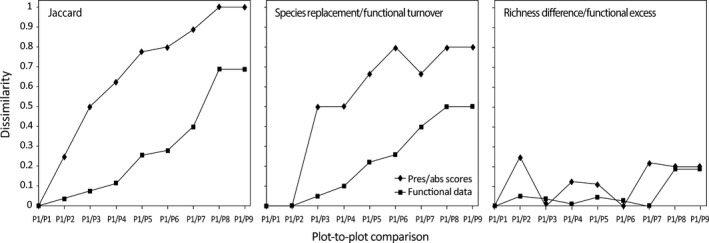
Graphical profiles showing the response to the simulated ecological gradient (Table 1) of the compositional (presence/absence scores only) Jaccard dissimilarity index, its functional generalization and their additive components, species replacement/functional turnover, and richness difference/functional excess. Plot *P1* is compared to itself and to all other plots in Table 1.

Figure 1 shows the graphical comparison between the compositional (presence/absence) and the functional versions of the Jaccard dissimilarity, together with their additive components, species replacement/functional turnover, and richness difference/functional excess.

In agreement with requirement (1), for all indices, the comparison of plot *P1* with itself results in zero dissimilarity. The graphical profiles of the compositional and functional versions of *J* and *J*_Repl_ both show an increasing pattern, which reflects the taxonomic and functional turnover along the simulated gradient of Table 1. By contrast, the graphical profiles of both versions of *J*_Rich_ show a more irregular pattern reflecting differences in species richness and functional excess between plot *P1* and all other plots.

**Figure 2:**
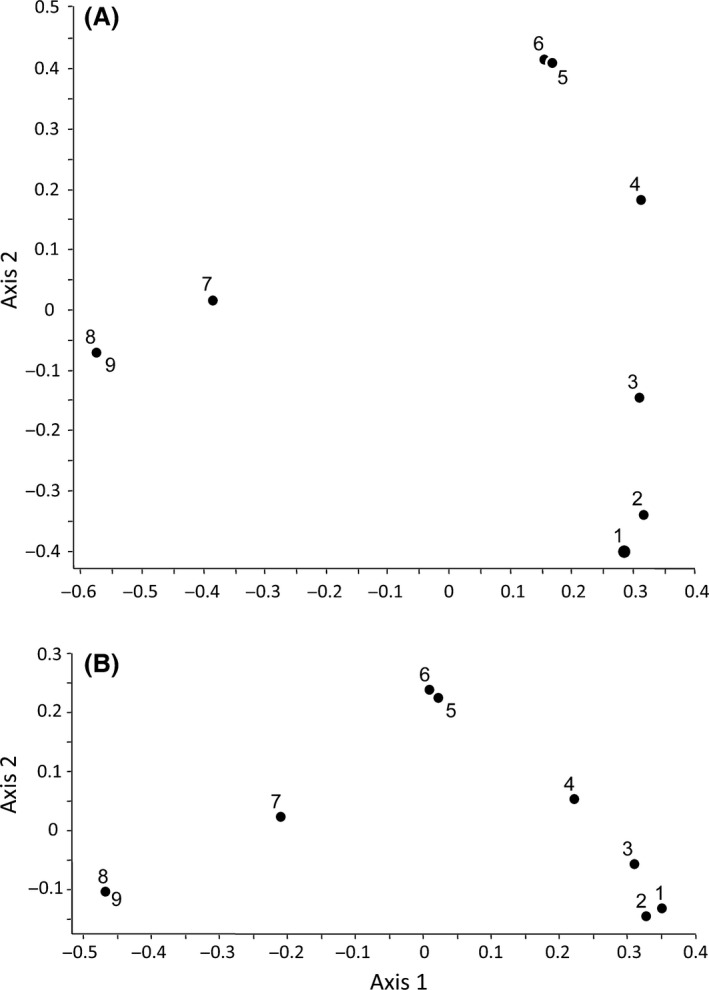
Principal coordinates ordinations of the artificial presence/absence scores in Table 1 based on (A) the traditional Jaccard index and (B) the generalized Jaccard index. Note the obvious detrending effect of incorporating functional information in the analysis.

The two ordinations of the nine sites are shown in Figure 2. As expected, the ordination based on presence/absence scores exhibits a conspicuous horseshoe or arch effect, reflecting the simulated unimodal response of the species to the underlying gradient. The eigenvalues of the first three ordination axes were 44.5, 24.2, and 9.9, respectively. In the functional ordination, the arch is less pronounced, meaning that the replacement of species presences and absences by the functional species dissimilarities had a detrending effect on the gradient. This is shown by the greatly increased first eigenvalue (72.5) and the lower subsequent eigenvalues (14.8 and 4.4 for the second and the third axes, respectively).

## Discussion

In this study, we proposed a new family of functional dissimilarity measures based on the generalization of the matching/mismatching components *a*,* b*, and *c* of the traditional contingency table to include functional dissimilarities among species. Unlike most indices used to date (e.g., Rao 1982; Izsák and Price 2001; Chiu and Chao 2014; Pavoine and Ricotta 2014), which are expressed in terms of average or minimum functional distances between pairs of plots (or as a derived function of these distances), the operational part and the scaling factor of the proposed family of measures are both expressed in terms of species numbers.

This approach is flexible enough to enable practitioners to generalize a large number of existing presence/absence dissimilarity measures or to “construct” new measures depending on the context of their analyses. In addition, relaxing requirement (2) that the index should take the value one *only* for maximally distinct assemblages, it will be possible to build an even larger family of indices. Here, it is worth noting that while the values of the functional mismatching components *B* and *C* are by definition not higher than the values of their traditional presence/absence analogues *b* and *c*, this does not necessarily hold for their “combinations”; for example, the absolute difference $\left|B-C\right|$ can be larger than $\left|b-c\right|$. Accordingly, as shown in Figure 1, the value of the functional version of *J*_Rich_ can be larger than its presence/absence counterpart, meaning that in this case the differences in functional richness between both plots are larger than the corresponding differences in taxonomic richness. This complementarity of viewpoints brought by the compositional and the functional approaches is also important for ordination studies, because, as shown by the worked example, extreme horseshoe effects may be reduced when the analysis shifts from pure compositional dissimilarities to functional dissimilarities.

Apart from a few exceptions (e.g., the indices proposed by Izsák and Price 2001), most of the functional dissimilarity indices published so far were developed for abundance data (e.g., Champely and Chessel 2002; Schmera et al. 2009; Ricotta and Bacaro 2010; Baiser and Lockwood 2011; Chiu and Chao 2014; Pavoine and Ricotta 2014; Sonnier et al. 2014). Among them, the indices dealing solely with relative abundances can only be adapted to cope with presence and absence data by assuming that all *N* species present in a given plot have an abundance equal to 1/*N*. However, in some cases, the incorporation of these abundances in the dissimilarity functions contrasts with the idea that the presence/absence scores (usually 0 and 1) represent a binary variable for which 1 stands for presence and 0 stands for absence without any reference to species abundances.

On the contrary, the indices that can deal with absolute abundances (e.g., Chiu and Chao 2014; Pavoine and Ricotta 2014) can be directly used with presence/absence scores by assuming that all species have unit abundance when present in a plot. But, as far as we are aware, none of them were expressed in terms of species richness while respecting requirements (1) and (2); for example, the indices proposed by Chiu and Chao (2014) can be expressed in terms of species richness (using functional Hill numbers) but have a different definition of two maximally distinct plots, whereas the indices of Pavoine and Ricotta (2014) satisfy requirements (1) and (2) but are expressed in terms of average functional dissimilarity among species. All these measures thus complete each other into a multifaceted approach to the calculation of plot‐to‐plot dissimilarity.

Finally, although we proposed this new family of dissimilarity measures in a functional context, the proposed approach is aimed at summarizing the dissimilarity between pairs of plots based on any between‐species dissimilarity measure of choice. Therefore, the same approach can be extended to any other ecologically meaningful measure of dissimilarity among species, such as phylogenetic dissimilarities rescaled to the range [0–1]. This is a very desirable property of our approach as it allows to summarize relevant aspects of plot‐to‐plot dissimilarity from different, equally relevant, standpoints.

## Conflict of Interest

None declared.

## Supporting information


**Appendix S1.** Input data used for the artificial example.

**Appendix S2.** Interspecies dissimilarity matrix used for the calculation of the generalized functional dissimilarity indices of the artificial example.

**Appendix S3.** R scripts for the calculation of the dissimilarity indices.

**Appendix S4.** Manual associated with the R scripts.
