# Supplementary material for: A family of functional dissimilarity measures for presence and absence data
Source: Ecol Evol. 2016 Jul 5;6(15):5383–9. doi: 10.1002/ece3.2214 (PMC4984511; doi:10.1002/ece3.2214)
Supplement: Supplementary file 4 — Appendix S4. Manual associated with the R scripts. [file ECE3-6-5383-s004.docx]

**Appendix 4. Manual associated with the R scripts**

R functions “PADDis”, "DJac" and "Jac" for calculating the dissimilarity coefficients introduced in the main text.

This program is free software: you can redistribute it and/or modify it under the terms of the GNU General Public License <http://www.gnu.org/licenses/>.

**Disclaimer:** users of this code are cautioned that, while due care has been taken and it is believed accurate, it has not been rigorously tested and its use and results are solely the responsibilities of the user.

**Description:** given a matrix of *N* plots × *S* species’ incidence (0/1) values, and an object of class 'dist' containing the (functional) dissimilarities among species, the function PADDis calculates the matching/misatching components a, b, c, A, B, C, for each pair of plots. It can also calculate the coefficients J and S developed in the main text + other coefficients from the same family, see below.

**Dependencies:** none. However, the scripts were developed by modifying the function 'dist.binary' of the package ade4 (Dray & Dufour 2007).

**Usage:** PADDis (comm, dis, method = NULL, diag = FALSE, upper = FALSE)

DJac (comm, dis, diag = FALSE, upper = FALSE)

Jac (comm, diag = FALSE, upper = FALSE)

**Arguments**

| *comm* | A matrix of *N* plots × *S* species containing the incidence (0/1) of all species in the *N* plots. Columns are species and plots are rows |
| --- | --- |
| *dis* | An object of class 'dist' containing the (functional) dissimilarities among species |
| *method* | An integer between 0 and 5. If NULL the choice is made with a console message. See details. |
| *diag* | a logical value indicating whether the diagonal of the distance matrix should be printed by ‘print.dist’ |
| *upper* | a logical value indicating whether the upper triangle of the distance matrix should be printed by ‘print.dist’ |

**Details:** If method=0, then the function PADDis returns 6 matrices corresponding to the a, b, c, A, B, and C values per pair of plots. Otherwise, it returns an object of class 'dist' corresponding to the dissimilarities among plots calculated with the following formulas:

$\frac{B+C}{a+b+c}$ # generalized Jaccard dissimilarity, with method = 1

$\frac{B+C}{2a+b+c}$# generalized Sørensen dissimilarity, with method = 2

$\frac{2\left(B+C\right)}{a+2\left(b+c\right)}$ # generalized Sokal & Sneath dissimilarity, with method = 3

$\frac{\sqrt{A+B}\sqrt{A+C}-A}{\sqrt{a+b}\sqrt{a+c}}$ # generalized Ochiai dissimilarity, with method = 4

$\frac{\min\left\{B,C\right\}}{a+\min\left\{b,c\right\}}$ # generalized Simpson dissimilarity, with method = 5

DJac and Jac use the additive decomposition of the Jaccard index. DJac takes into account the (functional) dissimilarities among species while Jac doesn't. Formulas used by DJac thus are:

$$J=\frac{B+C}{a+b+c}$$

$$J_{Repl}=\frac{2\min\left\{B,C\right\}}{a+b+c}$$

$$J_{Rich}=\frac{\left|B-C\right|}{a+b+c}$$

and those used by Jac are:

$$J=\frac{b+c}{a+b+c}$$

$$J_{Repl}=\frac{2\min\left\{b,c\right\}}{a+b+c}$$

$$J_{Rich}=\frac{\left|b-c\right|}{a+b+c}$$

**Example**

Load the data sets contained in Appendices 1 and 2 and name them Com and Dis, respectively. For that one can use the following instructions:

Com <- read.table(file.choose(), sep="\t", row.names=1, h=TRUE)

# here select Appendix 1.

Dis <- read.table(file.choose(), sep="\t", row.names=1, h=TRUE)

# here select Appendix 2.

Dis<- as.dist(Dis)

Load the functions contained in Appendix 3. For that, the following instructions can be used:

source(file.choose())

# here select Appendix 3

The following instructions were used to obtain Figure 1 in the main text:

J <- Jac(Com)

DJ <- DJac(Com, Dis)

plot(c(as.matrix(DJ$J)[1,]), ylab="Dissimilarity", xlab="Plot-to-plot comparison", pch=15, type="b", ylim=c(0,1), main="Jaccard")

lines(c(as.matrix(J$J)[1,]), type="b", pch=18)

legend("bottomright", legend=c("P/A scores", "functional data"), pch=c(15,18), lty=1)


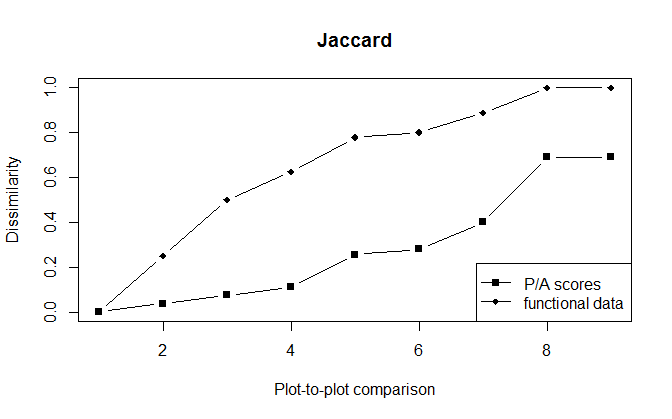


plot(c(as.matrix(DJ$JRepl)[1,]), ylab="Dissimilarity", xlab="Plot-to-plot comparison", pch=15, type="b", ylim=c(0,1), main="Species replacement")

lines(c(as.matrix(J$JRepl)[1,]), type="b", pch=18)

legend("bottomright", legend=c("P/A scores", "functional data"), pch=c(15,18), lty=1)


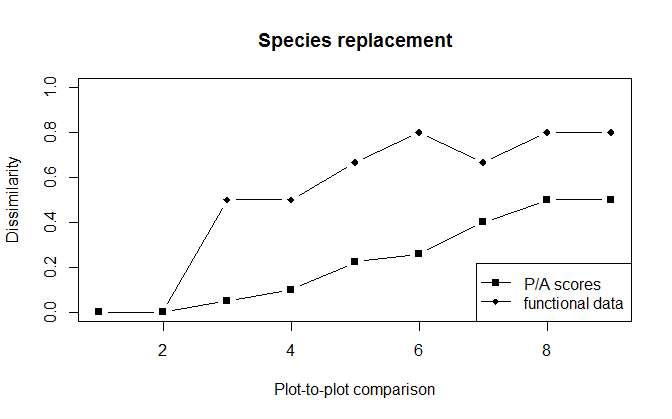


plot(c(as.matrix(DJ$JRich)[1,]), ylab="Dissimilarity", xlab="Plot-to-plot comparison", pch=15, type="b", ylim=c(0,1), main="Richness difference")

lines(c(as.matrix(J$JRich)[1,]), type="b", pch=18)

legend("topleft", legend=c("P/A scores", "functional data"), pch=c(15,18), lty=1)


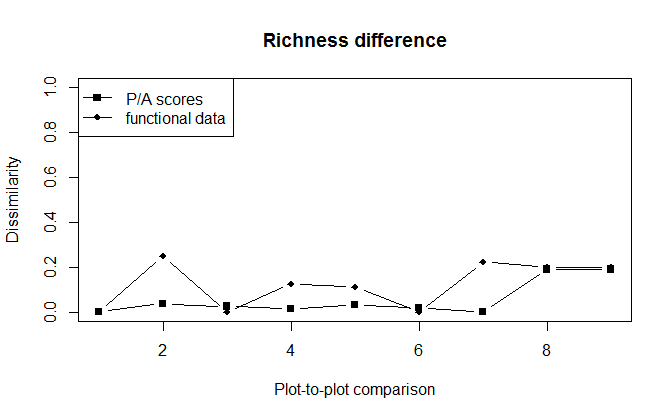


Use the following instruction to obtain all components:

**PADDis(Com, Dis)**

0 = list of all components (a, b, c, A, B, C)

1 = Jaccard index (1901)

d1 = (B+C)/(a+b+c)

2 = Czekanowski (1913) or Sorensen (1948)

d2 = (B+C)/(2*a+b+c)

3 = Sockal & Sneath (1963)

d3 = 2(B+C)/(a+2(b+c))

4 = Ochiai (1957)

d4 = [sqrt((A+B)(A+C))-A]/sqrt((a+b)(a+c))

5 = Simpson (1943)

d5 = min(B,C)/(a+min(b,c))

Select an integer (0-5): **0**

$a

P1 P2 P3 P4 P5 P6 P7 P8 P9

P1 6 6 4 3 2 2 1 0 0

P2 6 8 5 4 3 3 1 0 0

P3 4 5 6 4 3 3 1 0 0

P4 3 4 4 5 4 4 1 0 0

P5 2 3 3 4 5 5 2 1 1

P6 2 3 3 4 5 6 2 1 1

P7 1 1 1 1 2 2 4 3 3

P8 0 0 0 0 1 1 3 4 4

P9 0 0 0 0 1 1 3 4 4

$b

P1 P2 P3 P4 P5 P6 P7 P8 P9

P1 0 0 2 3 4 4 5 6 6

P2 2 0 3 4 5 5 7 8 8

P3 2 1 0 2 3 3 5 6 6

P4 2 1 1 0 1 1 4 5 5

P5 3 2 2 1 0 0 3 4 4

P6 4 3 3 2 1 0 4 5 5

P7 3 3 3 3 2 2 0 1 1

P8 4 4 4 4 3 3 1 0 0

P9 4 4 4 4 3 3 1 0 0

$c

P1 P2 P3 P4 P5 P6 P7 P8 P9

P1 0 2 2 2 3 4 3 4 4

P2 0 0 1 1 2 3 3 4 4

P3 2 3 0 1 2 3 3 4 4

P4 3 4 2 0 1 2 3 4 4

P5 4 5 3 1 0 1 2 3 3

P6 4 5 3 1 0 0 2 3 3

P7 5 7 5 4 3 4 0 1 1

P8 6 8 6 5 4 5 1 0 0

P9 6 8 6 5 4 5 1 0 0

$A

P1 P2 P3 P4 P5 P6 P7 P8 P9

P1 6.0 7.7 7.4 7.1 6.7 7.2 5.4 3.1 3.1

P2 7.7 8.0 8.6 7.9 7.5 8.1 7.0 4.2 4.2

P3 7.4 8.6 6.0 6.4 6.4 7.1 6.1 3.7 3.7

P4 7.1 7.9 6.4 5.0 5.2 6.0 5.8 4.2 4.2

P5 6.7 7.5 6.4 5.2 5.0 5.8 6.0 5.1 5.1

P6 7.2 8.1 7.1 6.0 5.8 6.0 6.7 5.8 5.8

P7 5.4 7.0 6.1 5.8 6.0 6.7 4.0 4.3 4.3

P8 3.1 4.2 3.7 4.2 5.1 5.8 4.3 4.0 4.0

P9 3.1 4.2 3.7 4.2 5.1 5.8 4.3 4.0 4.0

$B

P1 P2 P3 P4 P5 P6 P7 P8 P9

P1 0.0 0.0 0.2 0.5 1.3 1.3 1.8 4.4 4.4

P2 0.3 0.0 0.3 0.9 1.7 1.7 2.4 5.6 5.6

P3 0.4 0.1 0.0 0.4 0.8 0.8 1.3 4.1 4.1

P4 0.4 0.2 0.2 0.0 0.2 0.2 0.8 3.0 3.0

P5 1.0 0.8 0.8 0.6 0.0 0.0 0.5 2.1 2.1

P6 1.5 1.2 1.1 0.8 0.2 0.0 0.8 2.4 2.4

P7 1.8 1.6 1.6 1.4 0.5 0.5 0.0 0.6 0.6

P8 2.5 2.2 2.2 1.8 0.8 0.8 0.1 0.0 0.0

P9 2.5 2.2 2.2 1.8 0.8 0.8 0.1 0.0 0.0

$C

P1 P2 P3 P4 P5 P6 P7 P8 P9

P1 0.0 0.3 0.4 0.4 1.0 1.5 1.8 2.5 2.5

P2 0.0 0.0 0.1 0.2 0.8 1.2 1.6 2.2 2.2

P3 0.2 0.3 0.0 0.2 0.8 1.1 1.6 2.2 2.2

P4 0.5 0.9 0.4 0.0 0.6 0.8 1.4 1.8 1.8

P5 1.3 1.7 0.8 0.2 0.0 0.2 0.5 0.8 0.8

P6 1.3 1.7 0.8 0.2 0.0 0.0 0.5 0.8 0.8

P7 1.8 2.4 1.3 0.8 0.5 0.8 0.0 0.1 0.1

P8 4.4 5.6 4.1 3.0 2.1 2.4 0.6 0.0 0.0

P9 4.4 5.6 4.1 3.0 2.1 2.4 0.6 0.0 0.0

**References**

Dray, S. & Dufour, A.B. (2007) The ade4 package: implementing the duality diagram for ecologists. Journal of Statistical Software 22: 1-20.

Jaccard, P. (1900) Contribution au problème de l’immigration post-glaciaire de la flore alpine. Bulletin de la Société Vaudoise des Sciences Naturelles 36: 87–130.

Ochiai, A. (1957) Zoogeographic studies on the soleoid fishes found in Japan and its neighbouring regions. Bulletin of the Japanese Society of Scientific Fisheries 22: 526–530.

Simpson, G.G. (1943) Mammals and the nature of continents. American Journal of Science 241: 1–31.

Sokal, R.R. & Sneath, P.H.A. (1963) Principles of Numerical Taxonomy. W.H. Freeman, San Francisco.

Sørensen, T. (1948) A method of establishing groups of equal amplitude in plant sociology based on similarity of species content and its application in analysis of the vegetation on Danish commons. Biologiske Skrifter 5: 1–34.
